# Antifungal Effect of Cinnamon Bark Extract on the Phytopathogenic Fungus *Fusarium sporotrichioides*

**DOI:** 10.17113/ftb.62.04.24.8448

**Published:** 2024-12

**Authors:** Katarina Martinko, Eni Mioč

**Affiliations:** University of Zagreb Faculty of Agriculture, Division of Phytomedicine, Department of Plant Pathology, Svetošimunska 25, 10000 Zagreb, Croatia

**Keywords:** antifungal effect, aqueous cinnamon bark extract, *Fusarium sporotrichioides*, phytochemical tests, poisoned food technique, ultrasound-assisted extraction

## Abstract

**Research background:**

The use of plant extracts in the biological control of fungal plant diseases can reduce the use of fungicides and residues in food by effectively suppressing mycotoxigenic microorganisms. The focus of interest is therefore finding plant extracts that have antifungal properties and are not toxic to organisms, so that they can be used for the biological control of economically important phytopathogenic fungi such as *Fusarium*. Species of the genus *Fusarium* are considered economically important pathogenic fungi of numerous agricultural crops, which not only cause significant losses but also produce mycotoxins that reach consumers through food. One of the most important species of this genus is the species *Fusarium sporotrichioides*, which causes economically significant damage to a large number of agricultural crops.

**Experimental approach:**

In this laboratory study, the influence of aqueous cinnamon bark extract on the growth and development of the toxicogenic fungus *F. sporotrichioides* was investigated using the poisoned food technique. For the study, the aqueous extract of cinnamon bark was obtained by ultrasound-assisted extraction and the content of antifungal compounds was detected by phytochemical tests.

**Results and conclusions:**

The research results confirm a significant inhibition of the growth of the pathogen when grown individually on a potato-dextrose agar (PDA) medium with 3 and 5 % extract. The antifungal effect of the extract was demonstrated by microscopic analysis of the pathogen, which showed significant deformation of hyphae and a change in the mycelium colour after seven days of growth on medium with 5 % extract, resulting in a threefold higher inhibition of pathogen growth than growth on medium with 3 % extract. The microscopic changes also show a reduction in pathogen sporulation and a possible reduction in mycotoxin production. Phytochemical tests confirmed the presence of antifungal compounds in the extract.

**Novelty and scientific contribution:**

Based on the obtained results, the aqueous extract of cinnamon bark shows a fungistatic effect on the growth and development of *F. sporotrichioides*, which opens the possibility of continuing research of cinnamon compounds as potential compounds of future control agents for the suppression of fungi of the genus *Fusarium*.

## INTRODUCTION

Phytopathogenic fungi pose a threat to global food production, which leads to excessive use of fungicides and consequently to the development of pathogen resistance (*1*). Due to the modern problems in phytomedicine, the focus of interest is on the discovery of innovative methods for biological control of economically important phytopathogenic fungi using plant extracts that show antifungal properties and are ecologically acceptable. The importance of biological control is evidenced by studies investigating biological factors for this purpose, the number of which increased by 200 % in the 1990s (*2*). In addition to being ecologically acceptable and non-toxic to the environment, biological preparations have a mechanism of action that reduces the possibility of developing pathogen resistance (*3*).

The genus *Fusarium* is an economically important genus of fungi whose members are ubiquitous saprophytes, but also distinct polyphages because they have a wide range of host plants. Their economic importance is also reflected in the production of mycotoxins, *i.e*. secondary metabolites that are toxic, especially when they enter the human digestive system through food (*4*). Species of the genus *Fusarium* cause tracheomycosis, which causes plant wilt and fruit rot. The species of this genus are difficult to control because they spread through the soil, where they are held under unfavourable conditions by persistent chlamydospores (*5*). Among the important species of this genus is *Fusarium sporotrichioides* Sherb., which is the predominant cause of fusariosis on cereals, but also on various vegetables and fruits (*6*, *7*). Since the species *F. sporotrichioides* often occurs in a complex with other species of the genus *Fusarium* and causes an economically important disease - grain blight - treatment with fungicides is of great importance (*8*).

Today, the control of this pathogen is hindered by the limited number of fungicides available. According to the Croatian Ministry of Agriculture and the Phytosanitary Information System (*9*), the list of registered fungicidal preparations includes thirty-three preparations based on active substances from the triazole group, and most of the registrations expire at the end of 2024. According to recent studies (*8*), this fungus has hardly been researched despite its economic importance. It is worrying that it quickly develops resistance, especially to fungicides from the triazole group, which are dominant in suppressing fusariosis on cereals (*10*). It is known that the development of fungicide resistance is a consequence of a specific mechanism of action of fungicides, which is why efforts are being made to discover compounds that have a different mechanism of action on fungi (*11*). Among the biological factors used in the biocontrol of fungal pathogens, plant extracts that have antiinflammatory, antioxidant and antimicrobial properties stand out, which is why they are used in the food industry. One such plant is the species *Cinnamomum zeylanicum* Blume, from the bark of which cinnamon, a popular spice, is obtained (*12*). This tropical evergreen contains important antimicrobial compounds (cinnamaldehyde, cinnamic acid and eugenol), which have antifungal activity against phytopathogenic fungi (*13*). Research has shown that these compounds are found in different concentrations and in different parts of the plant (*14*), while Kyu *et al*. (*15*) have demonstrated the inhibitory effect of cinnamon extract on some species of the genus *Fusarium.* In order to extract essential compounds from plants, extraction as a biotechnological process is very important.

Due to the disadvantages of conventional extraction methods (high consumption of chemical solvents and energy), innovative green extractions are mainly used today. An interesting type of extraction is ultrasound-assisted extraction, which is attracting the interest of many researchers because of the efficient use of ultrasound energy, the mutual relationship between speed and high productivity and the absence of toxic solvents (*16*, *17*). Cerqueira Sales *et al.* (*18*) also support the use of nonchemical compounds in extraction with the aim of protecting human health and preserving the environment, as well as the use of green extraction to obtain plant extracts as the future of controlling mycotoxigenic fungi.

The main focus of this study is the investigation of the effect of an aqueous cinnamon bark extract *in vitro* on the growth and development of the toxicogenic fungus *F. sporotrichioides,* microscopic analysis of the anifungal effect of the extract on the pathogen microstructures and the phytochemical identification of the antifungal compounds in the extract.

## MATERIALS AND METHODS

### Plant material and fungal isolate

Organic cinnamon (*Cinnamomum zeylanicum*) powder was purchased from a local producer (Trgovina Farma 1, Zagreb, Croatia) and stored at 4 °C until use. An isolate of the pathogenic fungus *Fusarium sporotrichioides*, isolated from wheat grains and stored in the collection of the Department of Plant Pathology at the Faculty of Agriculture, University of Zagreb, Zagreb, Croatia, was used for the experiment. The fungus was molecularly identified to species level using a conventional polymerase chain reaction (PCR) method and sequenced at Macrogen Europe (Amsterdam, The Netherlands).

### Ultrasound-assisted extractions and preparation of extract

For the extraction, 10 g of cinnamon powder were mixed with 100 mL of sterile distilled water to perform an ultrasound-assisted extraction. To remove surface tension, 0.01 % surfactant (Tween 80; Sigma-Aldrich, Merck, Steinheim, Germany) was added to the water and powder solution. According to the modified method of Anal *et al.* (*19*), the extraction was carried out using water as a solvent and an ultrasonic bath (Emmi-D21; Emag, Steinheim, Germany) at 80 W and 40 kHz for 60 min at 50 °C. The obtained aqueous extract was centrifuged (centrifuge model 5425 R; Eppendorf, Hamburg, Germany) for 5 min (10 000×*g*) and then separated from the precipitate. The aqueous cinnamon bark extract (supernatant) was used at volume fractions of 1, 3 and 5 %, which was determined on the basis of preliminary tests and literature research (*20*, *21*).

### Poisoned food technique

The antifungal effect of aqueous cinnamon bark extract was tested using the poisoned food technique according to Ramaiah *et al.* (*22*).

A micellar disc (Ø 5 mm) of *F. sporotrichioides* was inoculated onto the previously poured potato-dextrose agar (PDA) medium (Sigma-Aldrich, Merck) and incubated in a climate chamber at 24 °C in the dark for 7 days. The extract at volume fractions of 1, 3 and 5 % was prepared by diluting stock solution and used for the experiments. To obtain a PDA medium with the extract at volume fractions of 1, 3 and 5 %, 1, 3 and 5 mL of extract were mixed with 9, 7 and 5 mL of PDA. The final volume was 10 mL per Petri dish. The extract was applied individually to the dissolved and partially cooled PDA medium. The solution was poured evenly into sterile plastic Petri dishes (Ø 8.5 cm). Micellar discs (Ø 5 mm) were cut from a 7-day-old culture of the *F. sporotrichioides* using a circular cutter and placed in the centre of the Petri dishes containing a medium with extract in different volume ratios (test Petri dishes) and medium without extract (control Petri dishes). The inoculated Petri dishes were incubated in a climate chamber at 24 °C in the dark. The experiment was set up in four variants and five repetitions. A total of 15 test and five control Petri dishes were used in the experiment.

### Microscopic analysis

The effect of the extract on the microstructures of *F. sporotrichioides* was investigated according to the modified method of Dėnė and Valiuškaitė (*23*) using a light microscope (BH2; Olympus, Tokyo, Japan) and a stereomicroscope (SZX7; Olympus) at 400× magnification.

To quantify the effect of the extract on the microstructure of the fungus, microscopic preparations were made with pathogen hyphae from a control Petri dish and from test variants with different volume ratios of the extract. The observed structural changes were photographed. The antifungal effect of the extract was quantified on the basis of the structural changes observed in the hyphae. The spores of the pathogen were also analysed in the same way. The microstructures were stained with lactophenol blue (Sigma-Aldrich, Merck) before microscopy.

### Phytochemical tests

Phytochemical tests were performed with an aqueous extract of cinnamon bark to determine the presence of antifungal compounds. To confirm the presence of flavonoids, alkaloids, terpenoids, tannins, phenols and quinones, the phytochemical tests according to modified methods of Parisa *et al*. (*24*), Adarsh *et al.* (*25*) and Pandey *et al.* (*26*) were carried out by adding reagents to the aqueous extract of cinnamon bark and the presence or absence of compounds was determined based on the occurrence of a specific chemical reaction. The addition of 200 μL chloroform (Sigma-Aldrich, Merck) and 200 μL sulfuric acid (T.T.T. d.o.o., Sveta Nedjelja, Croatia) to 1 mL extract resulted in a dark red colour in the presence of terpenoids. Mixing 500 μL hydrochloric acid (Sigma-Aldrich, Merck), 100 μL isoamyl alcohol (Sigma-Aldrich, Merck) and 0.2 g magnesium (T.T.T. d.o.o.) with the extract led to the appearance of an orange colour in the alcohol layer, indicating the presence of flavonoids. The addition of 0.2 g Fe(III) chloride (T.T.T. d.o.o.) to the aqueous extract resulted in a dark brown colour and indicated the presence of tannins. The addition of 0.3 g sodium hypochlorite (T.T.T. d.o.o.) resulted in a dark red colour, confirming the presence of quinone.

### ImageJ measurement

The photographs of the test and control Petri dishes after 7 days were processed with the computer program ImageJ (*27*) according to the modified method of Martinko *et al.* (*28*). Based on the mean values (cm^2^) of the micellar area of the pathogen, the inhibition index (I/%) was calculated and the antifungal effect of the aqueous cinnamon bark extract was quantified.

### Statistical analysis

The results are presented with mean values and standard deviations. Data were analysed using one-way analysis of variance (one-way ANOVA) and the differences between the treatments were evaluated using the Tukey's test (p≤0.05) in the statistical program SPSS v. 27 (*29*).

## RESULTS AND DISCUSSION

**Fig. 1:**
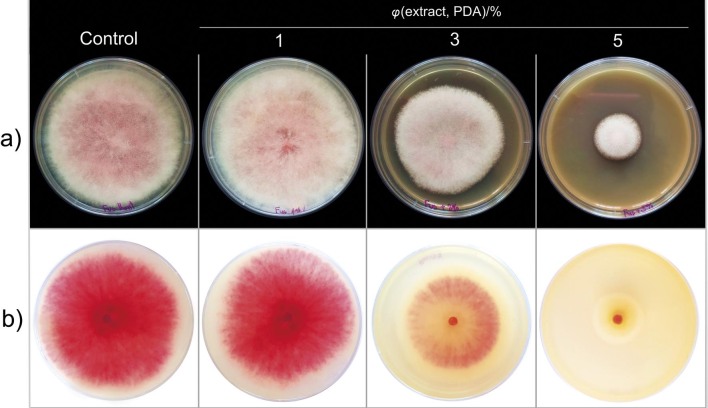
Effect of different volume ratios (*φ*) of aqueous extract of cinnamon bark on micellar development of the pathogen *Fusarium sporotrichioides* after 7 days on PDA: a) front side of the mycelium, and b) back side of the mycelium. PDA=potato-dextrose agar

The inhibition of mycelial growth at the lowest volume ratio of aqueous cinnamon bark extract (1 %) was not significant, while the growth of the fungus was significantly inhibited at higher volume ratios (3 and 5 %) compared to the control group. At the highest test volume ratio (5 %), the growth of *F. sporotrichioides* was suppressed three times more than the growth at 3 % extract in the medium (Table 1). Mvuemba *et al.* (*21*) reported the significant inhibitory effect of the same extract on mycotoxigenic species of the genus *Fusarium*, where the growth of the pathogen *F. sambucinum* was suppressed by 31 % after three days of growth on a medium with 5 % extract. Carmello *et al.* (*30*) confirmed the significant antifungal effect of 5 % extract when using the poisoned food technique on two strains of *F. oxysporum* f. sp. *lycopersici*, inhibiting the growth of one strain by 31 % and the other by 36 % under *in vitro* conditions. In addition, a significant reduction of pathogen spores was observed at the same 5 % extract. The results of Kowalska *et al.* (*31*) also confirmed the significant antifungal effect of the extract on the growth of *Botrytis cinerea* with a recorded micellar inhibition of 54 % when the 0.5 % extract was used, *i.e.* it was inhibited by 81 % when 1 % of the extract was added to the medium after 6 days. Comparing the results of other studies, the antifungal potential of the aqueous cinnamon bark extract appears to be selective depending on the pathogen isolate and strain (*30*), which shows a greater complexity in the mode of action of the active cinnamon compounds. In addition to phytopathogenic species, there are also studies on the significant antimycotic effects of aqueous cinnamon extract on human bacteria in dental medicine. In this study, the loss of the pink micellar pigment was observed with increasing extract content in the nutrient medium (Fig. 1). The correlation between the loss of the micellar red pigment of *Fusarium* sp. and toxin production was suggested by Duarte *et al.* (*32*), although they show that the mentioned correlation may depend on the pathogen strain. It is interesting that the red pigmentation of *Fusarium* species depends on the production of the pigment aurofusarin (*33*), which is also an important mycotoxin (*34*). Although complete micellar inhibition of the fungus was not observed even at the highest amount of extract (5 %), the observed loss of the red pigment may lead to the reduction of mycotoxins, which is more relevant for the suppression of the mycotoxigenic fungus *F. sporotrichioides*. This assumption is supported by the research of Xing *et al.* (*35*), where cinnamon oil almost completely (94 %) degraded the mycotoxin fumonisin, which is mainly produced by the species *F. verticillioides* and *F. proliferatum.*

**Table 1:** Antifungal effect of different volume ratios of cinnamon bark extract on mycelial growth area of the pathogen *Fusarium sporotrichioides* after 7 days

Parameter	Control	Test		
* φ*(extract, PDA)/%	0	1	3	5
*A*/cm^2^	(57.5±0.3)^a^	(55.7±0.8)^a^	(51.5±3.5)^b^	(17.2±1.9)^c^
I/%	0	3.1	10.4	70

Different letters in superscript indicate a statistically significant difference between mean values within the volume ratio values (Tukey’s test, p<0.05). *A*=pathogen growth area expressed as mean value±S.D. (*N*=5), I=inhibition index, PDA=potato-dextrose agar

**Fig. 2:**
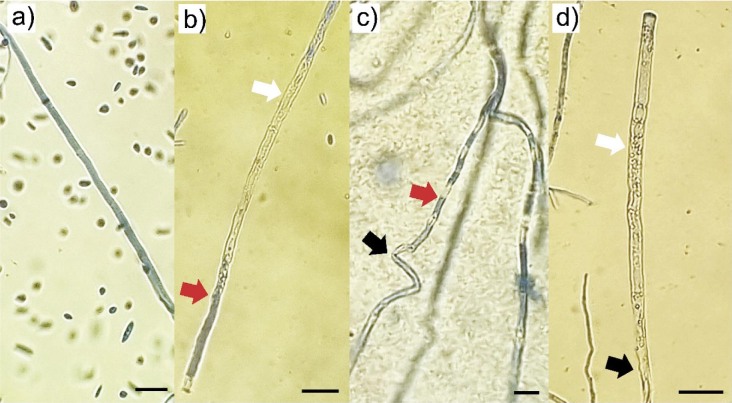
The effect of cinnamon bark extract (5 %) on the *Fusarium sporotrichioides* hyphae after 7 days: a) hyphae from the control, b-d) pathogen hyphal deformations: uneven staining with lactophenol blue indicating leakage of hyphal content (red arrows), vacuolisation of hyphal content (white arrows), twisting and collapse of hyphae (black arrows). Scale bar 50 μm, light microscope magnification 400×

**Fig. 3:**
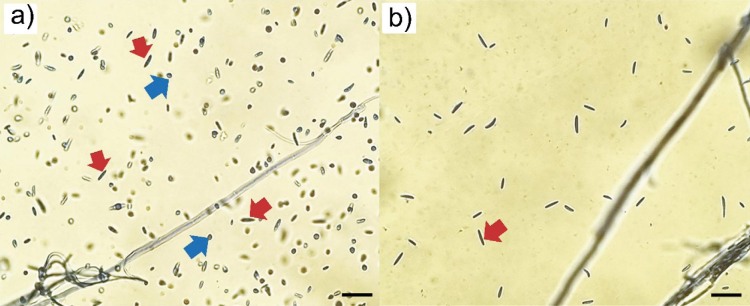
The effect of cinnamon bark extract (5 %) on the production of spores of the pathogen *Fusarium sporotrichioides* after 7 days: a) production of macroconidia (red arrows) and microconidia (blue arrows) in control variant without extract, and b) absence of microconidia production and development of macroconidia on the medium with 5 % extract. Scale bar 50 μm, light microscope magnification 400×

In addition to the macroscopic changes, significant deformations of the *F. sporotrichioides* hyphae were observed by light microscopy after 7 days of growth at the highest extract amount (5 %). Leakage of the hyphal content, vacuolisation of the content, twisting and collapse of the hyphae were observed, while the hyphae of the control variant were complete, turgid and regular (Fig. 2). The obtained results of the effect on the microstructures of pathogens are in agreement with the studies of Carmello *et al.* (*30*), whose results show similar effects of the cinnamon bark extract on the morphological characteristics of *F. oxysporum* hyphae, also at the same amount of 5 %. In addition to the morphological changes in hyphae, a decrease in sporulation and changes in the production of conidium types were observed. After 7 days, the fungus produced both types of conidia (macroconidia and microconidia) on the control variant without extract (Fig. 3a). On the medium containing 5 % of the extract, only the production of macroconidia was observed (despite the decrease in sporulation) (Fig. 3b). This situation needs to be investigated in more detail.

Many studies attribute the antifungal activity of cinnamon extract in the inhibition of *Fusarium* sp. to the presence of cinnamaldehyde compounds (*30*, *36*), cinnamic acid and eugenol (*13*, *22*) with synergistic effects with other listed phytochemical compounds (*30*). It is interesting to note that on the FRAC list (*37*), the flavonoid (cinnamic acid amide) extracted from the plant *C. zeylanicum* was included in the list of fungicidal substances due to its broad spectrum of activity, which includes the synthesis of cellulase - an enzyme that catalyses the process of cellulose decomposition, a compound that makes up the cell walls of fungi (*38*). The mentioned mechanism of action of the cinnamon compounds explains the results of the microscopic analysis. Obviously, the reported antifungal effects of cinnamon bark extract are attributed to the phytochemical compounds of cinnamon. The presence or absence of different phytoconstituents, *i.e.* tannins, saponins, flavonoids and terpenoids, was detected by phytochemical screening methods using different chemical reagents (*25*). The phytochemical tests in this study confirmed the presence of compounds such as tannins, flavonoids, terpenoids, alkaloids and quinones and the obtained results are in agreement with the research findings from the literature review (*24*-*26*).

## CONCLUSIONS

Based on the obtained results, when testing the effect of cinnamon bark extract on the phytopathogenic fungus *Fusarium sporotrichioides*, it was concluded that the extract has a significant fungistatic effect, especially on the medium with the highest amount of the extract (5 %). This is confirmed by the microscopic analysis of the microstructures of *F. sporotrichioides,* in which significant morphological changes in the mycelial pigment (fading), changes in sporulation, and deformation of the hyphae (collection, vacuolisation and outflow of hyphal contents) were found after pathogen growth on the extract with the highest test amount (5 %). The presence of phytochemical compounds with antifungal activity in the aqueous extract of cinnamon bark was tested after ultrasound-assisted extraction and the presence of phytochemical compounds with antifungal effect was demonstrated. The obtained results form the basis for future research on cinnamon extract, as the compounds have the potential to be used in the suppression of *Fusarium* species and the reduction of mycotoxins. In addition, more detailed studies under *in vitro* and *in vivo* conditions are needed.
